# Knowledge and Attitudes of Future Healthcare Professionals Toward Rare Diseases

**DOI:** 10.3389/fgene.2021.639610

**Published:** 2021-05-28

**Authors:** Jan Domaradzki, Dariusz Walkowiak

**Affiliations:** ^1^Department of Social Sciences and Humanities, Poznan University of Medical Sciences, Poznań, Poland; ^2^Department of Organization and Management in Health Care, Poznan University of Medical Sciences, Poznań, Poland

**Keywords:** genetic literacy, medical education, nursing students, physiotherapy students, medical students, future healthcare professional, rare diseases

## Abstract

Caring for patients suffering from a rare disease (RD) requires the special and combined efforts of different healthcare professionals, including nurses, physiotherapists and physicians. Nevertheless, Poland still lacks a national plan for RDs and the undergraduate and postgraduate education of future healthcare professionals on RDs is also inadequate. Thus, the aim of this study was to assess the awareness of RDs among nursing, physiotherapy and medical students in Poland. It shows that although 98% of respondents had heard of the term “rare disease,” most students had problems in defining the most common causes of RDs and their prevalence. Students also lacked basic knowledge about the healthcare system for RD patients in the country. While over 95% of future nurses, physiotherapists and physicians assessed their knowledge about RDs as insufficient or very poor, almost 92% of medical students, and 84% of physiotherapy and nursing students, did not feel prepared for caring for RD patients. Furthermore, although the vast majority of respondents declared eagerness to broaden their knowledge on RDs, only 45% of medical students, 76% of nursing students and 88% of physiotherapy students believed that RDs should be included into the medical curricula. Simultaneously, for most students the Internet was the prime source of information on RDs. It is concluded that as caring for RD patients requires a multidisciplinary approach, by identifying the gap in the education of future nurses, physiotherapists and physicians this study shows that there is an urgent need of better education about RDs among future healthcare professionals.

## Introduction

The European Union (EU) defines rare diseases (RDs) as chronically debilitating or life-threatening conditions of a prevalence of less than 5 per 10,000 persons (Eurordis, 2009). While there are approximately 27–36 million people in the EU suffering from as many as 6,000–8,000 different types of RDs (Montserrat and Taruscio, 2019; Czech et al., 2020) they require the special, combined efforts of different healthcare professionals, including physicians, physiotherapists, nurses, psychologists, dieticians or speech therapists. Thus, the implementation of such an interdisciplinary approach is strongly required because it can reduce the perinatal and early mortality of RD patients or increase their quality of life.

At the same time, while some 2–3 million persons suffer from RDs in Poland, available data suggests that still many RD patients remain without a diagnosis or proper treatment (Libura et al., 2016; Ministerstwo Zdrowia, 2019). Moreover, due to the minimal training of physicians on RDs, lack of information and awareness about RDs among healthcare professionals, poor communication among health providers and lack of standardized criteria for diagnosis, the search for a diagnosis and therapy often turns into an endless odyssey (Anderson et al., 2013; Black et al., 2015). This is important because misdiagnosis or late diagnosis results in delayed or many unnecessary treatments and hospitalizations, the worsening of an RD patient’s condition or his or her premature death. For all these reasons RDs are now widely recognized as an important medical, social and legal problem and an urgent public health issue (Schieppati et al., 2008). Consequently, in June 2009 the Council of the European Union recommended that by the end of 2013 all Member States (MS) should adopt a national plan or strategy for RDs (Council of the European Union, 2009). Moreover, throughout the years the EU has created an operational framework and coordinates several areas of common health policy in the field of RDs, including the classification and codification of RDs and orphan medicinal products, an ICD-10 revision and the creation of European Reference Networks or a European Platform for Rare Diseases registration (Moliner, 2010; Rodwell and Aymé, 2014, 2015; Moliner and Waligora, 2017; Khosla and Valdez, 2018; Montserrat and Taruscio, 2019; Czech et al., 2020). Nevertheless, while a lot has been done in the field of recommendations, in funding, and the reimbursement of orphan drugs in Europe (Kawalec et al., 2016; Zelei et al., 2016; Kolasa et al., 2018; Szegedi et al., 2018), still one of the most urgent areas in both the European and national health policies in the field of RDs is the medical education of healthcare students and professionals (Miteva et al., 2011; Budych et al., 2012; Anderson et al., 2013; Engel et al., 2013; Krajnović et al., 2013; Zurynski et al., 2017). Meanwhile, according to the EU recommendation, social and medical education on RDs should be one of the key areas of each national plan or strategy (Council of the European Union, 2009).

Nevertheless, although only a few MS, i.e., France and Spain, have fully implemented national plans/strategies for RDs according to the EU’s recommendations, and the vast majority have already created such plans (i.e., Belgium, Bulgaria, Cyprus, the Czech Republic, Denmark, Germany, Greece, Hungary, Ireland, Italy, Latvia, Lithuania, Portugal, Romania, the Slovak Republic, Slovenia, the Netherlands, and the United Kingdom), Poland is among the three EU countries, next to Malta and Sweden, that still have not adopted their national plans (Khosla and Valdez, 2018; Montserrat and Taruscio, 2019; Czech et al., 2020). However, according to the recent declaration of the Polish Ministry of Health, Poland should promulgate such a strategy by June 2020 (Ministerstwo Zdrowia, 2019). It is important because one of its strategic points is the improvement of undergraduate and postgraduate education on RDs by including lectures and seminars on RDs during the last 2 years of studies and by creating a system of postgraduate training offering mandatory specialization training sessions and specialized courses on RDs. Simultaneously, nowhere does the document specify or differentiate between the educational needs of undergraduate and graduate students. This is of key importance because previous research projects show that future healthcare professionals in the country lack general knowledge of RDs and there is an urgent need to raise the awareness on RD among medical students and to educate them about such diseases (Kopeć and Podolec, 2015; Jonas et al., 2017; Domaradzki and Walkowiak, 2019; Walkowiak and Domaradzki, 2020). Moreover, although RD patients require interdisciplinary approach, most previous studies focused on the knowledge and awareness on RDs among medical students and general practitioners (Miteva et al., 2011; Byrne, 2012; Kopeć and Podolec, 2015; Wolyniak et al., 2015; Medić et al., 2016; Jonas et al., 2017; Domaradzki and Walkowiak, 2019; Ramalle-Gómara et al., 2020). Meanwhile, although it is physicians who coordinate the process of caring for RD patients the role of other healthcare professionals, including nurses and physiotherapists, in managing of RDs is increasing, especially so that most RDs affect children. Consequently, there is also a need for enhancing genetic literacy on RDs among all healthcare professionals.

It is of special importance, because while medical, nursing and physiotherapy students in Poland receive classes in clinical genetics for at least two semesters, where they are taught about some genetic diseases (i.e., PKU, CF, sickle cell anemia, Huntington disease, Pompe disease, Niemann-Pick disease), the methods and types of materials used in genetic laboratory diagnostics, they do not receive special training in clinical genetics ambulatories. Neither do they receive any particular course on RDs. Moreover, also postgraduate specialization trainings for physicians, nurses and physiotherapists supervised by the Polish Minister of Health give little or no such information on RDs. For example, postgraduate specialization courses in nursing offer only a 1-h lecture dedicated to the National Rare Disease Plan and education in the field of RD. However, neither qualification courses nor specialized courses give any information on caring for RD patients (Walkowiak and Domaradzki, 2020). Thus, the objective of our study was to assess the knowledge and awareness of RDs among nursing, physiotherapy and medical students of the Poznan University of Medical Sciences (PUMS), Poland.

## Materials and Methods

The study was conducted between October and December 2019 among nursing, physiotherapy and medicine students of the PUMS. However, because it takes 6 years to complete medical studies in Poland, while both nursing and physiotherapy programs are five years long (3-year Bachelor’s degree and 2-year Master’s degree), this research was conducted among *students* during the 2 final years of their studies. The student participants were recruited during regular classes. A standard questionnaire was used, comprising the topics based on the literature review and the study’s aim. The detailed description of the questionnaire and the method used for its development has been described elsewhere (Domaradzki and Walkowiak, 2019; Walkowiak and Domaradzki, 2020). Briefly, the questionnaire comprised of 28 questions: 22 items referred to respondents’ knowledge of and attitudes toward RD and 6 questions that addressed their demographic data, and was divided into four sections. The first questions regarded students’ basic knowledge on RDs, such as their definition, etiology and estimated prevalence of RDs worldwide, in the EU and in Poland. Students were also asked to indicate RDs from a list comprising twenty eight diseases: eighteen RDs and 10 more common disorders. We have chosen RDs that are either commonly known conditions (i.e., progeria, Huntington disease, hemophilia or sickle cell anemia) or are included into medical curricula (i.e., Pompe disease, Gaucher disease, Niemann-Pick disease, phenylketonuria). The second section included questions regarding organizational issues, including the Polish rare disease policy and the orphan drug reimbursement system. The third section were questions on students’ education about RDs and their self-assessment of the knowledge and competence in the field of RDs. The last section of the questionnaire included questions concerning students’ demographic characteristics.

The process of elaborating the questionnaire itself followed the guidelines of the European Statistical System (Eurostat, 2005). A total of 18 subjects were involved in the development of the questionnaire (five nursing students, five medical students, five physiotherapy students, one geneticist, one sociologist and one statistician) who elaborated a list of important issues on RDs, which resulted in developing a questionnaire which was assessed by five external reviewers (one nurse, one physiotherapist, one physician, one geneticist and one sociologist). Second, the questionnaire was pre-tested in face-to-face meetings with another ten nursing, physiotherapy and medicine students, which resulted in the reformulation of four questions. The final version of the questionnaire was again evaluated by another five external reviewers from the same specialties. After receiving final approval, the survey was distributed to all the students who had volunteered. Ethics approval was obtained from the PUMS Bioethics Committee (1018/18). Informed consent was obtained from all individual participants included in the study.

The data collected in the questionnaires were verified and checked for completeness, quality and consistency. Then, they were coded and exported into the statistical packages JASP (Version 0.12.2) and STATISTICA 13.1 (TIBCO, Palo Alto, United States). The results were presented as descriptive statistics. A Likelihood Ratio Chi-square was used to assess the differences in the distribution of answers among the groups. The odds ratio (OR) was calculated to compare one group of students according to different characteristics based on their opinions in relation to other groups of students. The 95% confidence interval (95% CI) was calculated to estimate the precision of the OR. A 5% level of significance was used for all hypothesis tests.

## Results

Out of all the 862 students approached, 654 (75.9%) completed the questionnaire (Table 1). 208 students who refused to participate did so because they either lacked interest in the study or were unwilling to discuss their knowledge on RDs. The feedback on surveys from the nursing students (NS) was 113/120 (94.2%), from the physiotherapy students (PS)—173/219 (79.0%) and from the medical students (MS)—368/523 (70.4%). The sample consisted of 463 females (70.8%) and 191 males (29.2%), all of Polish origin. 43 students declared having a person suffering from a RD in their family (18.4%). One physiotherapy student suffered from a RD herself, although no such question was asked in the questionnaire.

**TABLE 1:** Socio-demographic characteristics of students.

Characteristics	N (%)		
	Nursing students	Physiotherapy students	Medical students
**Year of study**			
4th nursing and physiotherapy students/5th medical students	56 (49.6)	86 (49.7)	201 (54.6)
5th nursing and physiotherapy students/6th medical students	57 (50.4)	87 (50.3)	167 (45.4)
**Gender**			
Female	104 (92)	136 (78.6)	223 (60.6)
Male	9 (8)	37 (21.4)	145 (39.6)
**Have you ever met a person suffering from RD**			
Yes	38 (33.6)	83 (48)	268 (72.8)
No	54 (47.8)	46 (26.6)	65 (17.7)
I do not know	21 (18.6)	44 (25.4)	35 (9.5)
**Is anyone in your family suffering from RD?**			
Yes	4 (3.5)	14 (8.1)	25 (6.8)
No	99 (87.6)	141 (81.5)	292 (79.4)
I do not know	10 (8.9)	18 (10.4)	47 (12.8)

More than 98% of students in our study groups declared having heard the term “rare disease” (Table 2). However, while almost 90% of the medical students and nearly 70% of the physiotherapy students knew what was the most common cause of RDs, among the nursing students it was only 60%. Nevertheless, only about 30% in each group correctly estimated the percentage of RDs of genetic origin. At the same time, physiotherapy students’ estimates of the number of RD patients worldwide and in Poland were the most accurate, although only 14% of them gave the correct answers. Similarly, while most students knew that RDs affect mostly the newborns and children, very few students knew the correct prevalence of RDs (NS = 7.1%, PS = 6.4, MS = 11.7) and the number of RDs (NS = 6.2%, PS = 13.9%, MS = 10.9%).

**TABLE 2:** Students’ knowledge about rare diseases.

	N (%)			Comparison between groups p		
	Nursing students	Physiotherapy students	Medical students	NS vs. PS	NS vs. MS	PS vs. MS
**Have you ever heard the term “rare diseases”?**						
Yes	107 (94.7)	171 (98.8)	365 (99.2)	0.04	0.005	ns
No	6 (5.3)	2 (1.2)	3 (0.8)			
Rare disease is the one that affects less than:						
1 person in 1,000	17 (15)	31 (17.9)	25 (6.8)			
**1 person in 2,000**	**8 (7.1)**	**11 (6.4)**	**43 (11.7)**	ns	ns	0.04
1 person in 3,000	1 (0.9)	4 (2.3)	11 (3)			
1 person in 5,000	5 (4.4)	23 (13.3)	12 (3.2)			
1 person in 10,000	52 (46)	92 (53.2)	213 (57.9)			
I do not know	30 (26.6)	12 (6.9)	64 (17.4)			
**What is the estimated number of rare diseases?**						
100–500	17 (15)	24 (13.9)	27 (7.3)			
1,000–2,000	37 (32.7)	50 (28.9)	52 (14.1)			
3,000–5,000	14 (12.4)	33 (19.1)	49 (13.3)			
**6,000–8,000**	**7 (6.2)**	**24 (13.9)**	**40 (10.9)**	0.03	ns	ns
9,000–10,000	6 (5.3)	11 (6.3)	19 (5.2)			
Over 10,000	9 (8)	18 (10.4)	126 (34.2)			
I do not know	23 (20.4)	13 (7.5)	55 (15)			
**At what age group do rare diseases most frequently appear?**						
Newborns	29 (25.7)	54 (31.2)	172 (46.7)			
**Children**	**42 (37.2)**	**63 (36.4)**	**99 (26.9)**	ns	0.04	ns
Adolescents	3 (2.6)	6 (3.5)	10 (2.7)			
Adults	6 (5.3)	7 (4)	8 (2.2)			
They are present in all age groups equally	21 (18.6)	38 (22)	40 (10.9)			
I do not know	12 (10.6)	5 (2.9)	39 (10.6)			
**How many people suffer from rare diseases worldwide?**						
10–15,000,000	23 (20.3)	27 (15.6)	66 (17.9)			
50–75,000,000	35 (31)	38 (22)	68 (18.5)			
100–150,000,000	15 (13.3)	51 (29.5)	76 (20.7)			
200–250,000,000	5 (4.4)	16 (9.2)	32 (8.7)			
**300–350,000,000**	**7 (6.2)**	**25 (14.4)**	**35 (9.5)**	0.02	ns	ns
Over 500,000,000	1 (0.9)	1 (0.6)	28 (7.6)			
I do not know	27 (23.9)	15 (8.7)	63 (17.1)			
**How many people suffer from rare diseases in Poland?**						
500–1,000	22 (19.5)	17 (9.8)	24 (6.5)			
10–15,000	28 (24.8)	43 (24.9)	72 (19.6)			
50–75,000	17 (15.1)	37 (21.4)	61 (16.6)			
100–150,000	11 (9.7)	24 (13.9)	67 (18.2)			
300–500,000	5 (4.4)	17 (9.8)	56 (15.2)			
1 000 000	2 (1.8)	3 (1.7)	6 (1.6)			
**2–3,000,000**	**4 (3.5)**	**24 (13.9)**	**18 (4.9)**	0.002	ns	0
Over 5,000,000	0	0	6 (1.6)			
I do not know	24 (21.2)	8 (4.6)	59 (16)			
**What is the most common cause of rare diseases?**						
Infectious and bacterial	5 (4.4)	12 (6.9)	2 (0.5)			
**Genetic**	**67 (59.3)**	**116 (67)**	**330 (89.6)**	ns	0	0
Autoimmune	26 (23)	36 (20.8)	13 (3.5)			
Mitochondrial	3 (2.7)	7 (4)	4 (1.1)			
Environmental	1 (0.9)	4 (2.3)	2 (0.5)			
I do not know	11 (9.7)	16 (4.6)	17 (4.6)			
**What percentage of rare diseases are of genetic origin?**						
5–10%	19 (16.8)	17 (9.8)	11 (3)			
20%	27 (23.9)	50 (28.9)	54 (14.7)			
50%	28 (24.8)	48 (27.7)	135 (36.7)			
**80%**	**30 (26.5)**	**52 (30.1)**	**131 (35.6)**	ns	ns	ns
100%	0	1 (0.6)	18 (4.9)			
I do not know	9 (8)	5 (2.9)	19 (5.1)			

**Correct answers are written in boldface.**

From the presented list of 28 diseases (including 18 RDs), students chose those they considered to be rare diseases (Table 3). Among medical and nursing students the most frequently recognized RDs were Pompe disease, Gaucher disease and Niemann-Pick disease, while among physiotherapy students it was Pompe disease, Niemann-Pick disease and Fragile X syndrome. On the other hand, in all three groups the most common disease that was mistaken with RDs turned out to be Munchausen syndrome. The statistical analysis of the frequency of indications for specific RDs in individual groups of students revealed the existence of many statistically significant differences. Future physicians identified more RDs from the list correctly more frequently than nursing students, although in the case of non-RDs it was exactly the opposite: nursing students indicated them as RDs less often than medical students did. Similarly, medical students selected RDs correctly more often than physiotherapy students, although in the case of acromegaly, Fragile X syndrome and Marfan syndrome, the opposite was the case. On the other hand, physiotherapy students scored higher than nursing students. The results of the analysis are ambiguous and indicate evident knowledge gaps in all student groups. Despite the fact that on average future doctors gave the best answers, still they also indicated most of non-RDs, including acquired immunodeficiency syndrome as a RD most often.

**TABLE 3:** Which of the following diseases are considered to be rare in Poland?

	N (%)			Comparison between groups p		
	Nursing students	Physiotherapy students	Medical students	NS vs. PS	NS vs. MS	PS vs. MS
**Sickle cell anemia**	6 (5.3)	33 (19.1)	48 (13)	0	0.01	ns
**Cystic fibrosis**	15 (13.3)	48 (27.7)	87 (23.6)	0.003	0.009	ns
**Acromegaly**	19 (16.8)	56 (32.4)	61 (16.6)	0.003	ns	0.004
**Hemophilia**	9 (8)	20 (11.6)	85 (23.1)	ns	0	0.001
Down syndrome	2 (1.8)	2 (1.2)	19 (5.2)	ns	ns	0.01
**Niemann-Pick disease**	56 (49.6)	86 (49.7)	242 (65.8)	ns	0	0
Halitosis	12 (10.6)	32 (18.5)	97 (26.4)	ns	0	0.04
Glaucoma	0	3 (1.7)	16 (4.4)	ns	0.003	ns
**Progeria**	32 (28.3)	60 (34.7)	206 (56)	ns	0	0
**Neurofibromatosis**	10 (8.9)	52 (30.1)	110 (29.9)	0	0	ns
**Craniodiaphyseal dysplasia**	18 (15.9)	45 (26)	158 (42.9)	0.04	0	0
Cerebral palsy	6 (5.3)	1 (0.6)	30 (8.2)	0.01	ns	0
Fibromyalgia	38 (33.6)	30 (17.3)	121 (32.9)	0.01	ns	0
**Huntington disease**	36 (31.9)	64 (37)	169 (45.9)	ns	0.007	0.05
**Duchenne muscular dystrophy**	35 (31)	49 (28.3)	162 (44)	ns	0.01	0
Acquired immunodeficiency syndrome	6 (5.3)	26 (15)	87 (23.6)	0.007	0	0.02
Munchausen syndrome	59 (52.2)	85 (49.1)	186 (50.5)	ns	ns	ns
**Mucopolysaccharidoses**	19 (16.8)	51 (29.5)	176 (47.8)	0	0.01	0
**Achondroplasia**	21 (18.6)	33 (19.1)	102 (27.7)	ns	0.05	0.03
Crohn’s disease	12 (10.6)	54 (31.2)	17 (4.6)	0	0.03	0
**Pompe disease**	58 (51.3)	94 (54.3)	268 (72.8)	ns	0	0
**Gaucher disease**	56 (49.6)	81 (46.8)	254 (69)	ns	0	0
**Fragile X syndrome**	48 (42.5)	82 (47.4)	140 (38)	ns	ns	0.04
**Marfan syndrome**	43 (38.1)	80 (46.2)	109 (29.6)	ns	ns	0
Schizophrenia	1 (0.9)	1 (0.6)	8 (2.2)	ns	ns	ns
Alzheimer’s disease	1 (0.9)	1 (0.6)	15 (4.1)	ns	ns	0.01
**Osteogenesis imperfecta**	18 (15.9)	54 (31.2)	202 (54.9)	0.005	0	0
**Phenylketonuria**	14 (12.4)	54 (31.2)	155 (42.1)	0	0	0.01

**ns, not significant.**

Physiotherapy students were of the opinion that RDs constitute a serious public health issue more often than medical and nursing students (Table 4). However, more than 75% of medical students also believed that RDs should be prioritized. The difference between all three groups was statistically significant (*p* = 0.000). At the same time, approximately 60% of all students falsely believed that there is a central register of RD patients in Poland. Similarly, more than 40% of nursing students and almost 60% of physiotherapy and medical students believed that orphan drugs are reimbursed in Poland, which is also not true. Simultaneously, it was medical students who knew the name of the European website providing information about RD and orphan drugs best (20.6% vs. 11% PS and 0.9% NS). Finally, approximately one-third of each group knew how many RDs can be treated with drugs.

**TABLE 4:** Students’ knowledge about the healthcare system for RD patients.

	N (%)			Comparison between groups p		
	Nursing students	Physiotherapy students	Medical students	NS vs. PS	NS vs. MS	PS vs. MS
**What is the name of the European website providing information about RD and orphan drugs?**						
Rare Disease Foundation	3 (2.7)	19 (11)	10 (2.7)			
NORD	4 (3.5)	7 (4)	4 (1.1)			
EURORDIS	11 (9.7)	30 (17.3)	26 (7.1)			
R.A.R.E	5 (4.4)	15 (8.7)	28 (7.6)			
**Orphanet**	**1 (0.9)**	19 (11)	**76 (20.6)**	0	0	0.004
Global Genes	1 (0.9)	4 (2.3)	6 (1.6)			
I do not know	88 (77.9)	79 (45.7)	222 (60.3)			
**Is there a central register of RD patients in Poland?**						
Yes	64 (56.6)	108 (62.4)	228 (61.9)			
**No**	**10 (8.9)**	**26 (15)**	**15 (4.1)**	ns	ns	0
I do not know	39 (34.5)	39 (22.6)	125 (34)			
**What percentage of rare disease can be treated with drugs?**						
0%	6 (5.3)	5 (2.9)	1 (0.3)			
**5%**	**30 (26.5)**	**58 (33.5)**	**117 (31.8)**	ns	ns	ns
10%	22 (19.5)	39 (22.5)	67 (18.2)			
15%	16 (14.2)	28 (16.2)	69 (18.7)			
20%	11 (9.7)	28 (16.2)	33 (9)			
50%	1 (0.9)	9 (5.2)	5 (1.4)			
I do not know	27 (23.9)	6 (3.5)	76 (20.6)			
**Are orphan drugs reimbursed in Poland?**						
Yes	2 (1.8)	6 (3.5)	7 (1.9)			
**Yes, some**	**50 (44.3)**	**101 (58.4)**	**220 (59.8)**	0.02	0.004	ns
No	15 (13.3)	46 (26.6)	41 (11.1)			
I do not know	46 (40.7)	20 (11.5)	100 (27.2)			
**Do RDs constitute a serious public health issue?**						
Absolutely yes	29 (25.7)	77 (44.5)	60 (16.3)			
Yes	67 (59.3)	77 (44.5)	224 (60.9)			
No	6 (5.3)	15 (8.6)	58 (15.8)			
Definitely not	1 (0.9)	2 (1.2)	1 (0.3)			
I do not know	10 (8.8)	2 (1.2)	24 (6.5)			

*Correct answers are written in boldface.*

Some significant differences between groups of nursing, physiotherapy and medical students were observed. While only four nursing students assessed their knowledge about RDs as very good, there was no such person among medical and physiotherapy students (Tables 5, 6). On the other hand, there was a statistically significantly difference between nursing students who rated their knowledge as very poor and other students (LR, *p* = 0.01). Despite this difference, in each group at least 95% of students assessed their knowledge on RDs as insufficient or very poor. Moreover, almost 92% of medical students, and 84% of physiotherapy and nursing students did not feel prepared for caring for RD patients. In turn, 11% of physiotherapy students found themselves prepared to care for such patients. While the vast majority of students declared their eagerness to broaden their knowledge on RDs, only 45% of future doctors believed that such a topic should be included into the medical curricula. In contrast, among nursing students it was 76% and among physiotherapy students 88%. These declarations of future doctors were, statistically, significantly different from those of the nursing and physiotherapy students. While some medical students did not feel prepared to care for RD patients and declared their wish to broaden their knowledge, they did not see the need to include the RDs topics into the curricula. Interestingly, for all three groups of students the Internet was the most important source of information on RDs, although medical students also pointed out the mandatory and elective courses at the university.

**TABLE 5:** Students’ self-assessment of their knowledge about RDs, split faculty.

	N (%)		
	Nursing students	Physiotherapy students	Medical students
**How would you rate your knowledge about rare diseases?**			
Very good	4 (3.5)	0	0
Fair enough	2 (1.8)	9 (5.2)	18 (4.9)
Insufficient	46 (40.7)	97 (56.1)	207 (56.2)
Very poor	61 (54)	67 (38.7)	143 (38.9)
**Do you feel prepared for caring for a patient with a rare disease?**			
Definitely	0	2 (1.2)	1 (0.3)
Rather yes	3 (2.7)	17 (9.8)	17 (4.6)
Rather not	51 (45.1)	95 (54.9)	152 (41.6)
Definitely not	44 (38.9)	50 (28.9)	185 (50.3)
I do not know	15 (13.3)	9 (5.2)	13 (3.2)
**Would you like to broaden your knowledge about rare diseases?**			
Yes	94 (83.2)	147 (85)	272 (73.9)
No	3 (2.7)	14 (8.1)	44 (12)
I do not know	16 (14.2)	12 (6.9)	52 (14.1)
**Do you think that there should be a mandatory course on rare diseases in medical curricula?**			
Definitely yes	26 (23)	55 (31.8)	23 (6.2)
Rather yes	60 (53.1)	97 (56.1)	145 (39.4)
Rather not	12 (10.6)	16 (9.2)	142 (38.6)
Definitely not	2 (1.8)	0	28 (7.6)
I do not know	13 (11.5)	5 (2.9)	30 (8.2)
**Did you/do you have any classes about rare diseases during your studies?**			
Yes	36 (31.9)	89 (51.5)	282 (76.6)
No	60 (53.1)	76 (43.9)	63 (17.1)
I do not know	17 (15)	8 (4.6)	23 (6.3)
**Where do you/did you get your knowledge about RDs from?**			
Mandatory courses at the university	12 (10.6)	56 (32.4)	188 (51.1)
Facultative courses at the university	9 (8)	20 (11.6)	82 (22.3)
Scientific literature and research	15 (13.3)	16 (9.3)	72 (19.6)
Scientific conferences, symposia	7 (6.2)	10 (5.8)	36 (9.8)
Internet	62 (54.9)	92 (53.2)	216 (58.7)
Other	3 (2.7)	2 (1.2)	9 (2.5)
I do not search for such information	27 (23.9)	30 (17.3)	42 (11.4)

**TABLE 6:** The odds ratio of different aspects of nursing, physiotherapy and medical students’ self-assessment.

**Odds of being of the opinion that there should be a mandatory course on rare diseases in medical curricula**			
	Nursing students vs. physiotherapy students	Nursing students vs. medical students	Physiotherapy students vs. medical students
OR	0.44	3.79	8.62
95%CI	0.24–0.83	2.35–6.12	5.23–14.21
p	**0.005**	**0**	**0**
**Odds of being interested in broadening knowledge about RDs**			
	Nursing students vs. physiotherapy students	Nursing students vs. medical students	Physiotherapy students vs. medical students
OR	0.88	1.75	2.00
95%CI	0.46–1.67	1.01–3.01	1.24–3.22
p	0.41	**0.02**	**0.002**
**Odds of being of the opinion that RDs constitute a serious public health issue**			
	Nursing students vs. physiotherapy students	Nursing students vs. medical students	Physiography students vs. medical students
OR	0.70	1.65	2.37
95%CI	0.35–1.41	0.93–2.92	1.39–4.05
p	0.16	0.04	**0.001**
**Odds of feeling prepared for caring for a patient with RD**			
	Nursing students vs. physiotherapy students	Nursing students vs. medical students	Physiotherapy students vs. medical students
OR	0.22	0.53	2.40
95%CI	0.06–0.77	0.15–1.83	1.23–4.70
p	**0.008**	0.16	**0.005**
**Odds of reporting not searching information about RDs**			
	Nursing students vs. physiotherapy students	Nursing students vs. medical students	Physiotherapy students vs. medical students
OR	1.50	2.40	1.60
95%CI	0.83–2.69	1.40–4.11	0.96–2.67
p	0.09	**0.001**	0.03
**Odds of reporting getting get knowledge about RDs from mandatory course at the university**			
	Nursing students vs. physiotherapy students	Nursing students vs. medical students	Physiotherapy students vs. medical students
OR	0.25	0.11	0.45
95%CI	0.13–0.49	0.06–0.21	0.31–0.65
*p*	**0**	**0**	**0**

*Statistically signifcant diferences are written in boldface.*

As for the year of studies and the faculty, no significant differences between nursing and physiotherapy students were found, whereas among medical students the last year students felt better prepared to care for RD patients than those studying at the penultimate year (Table 7). Nevertheless, sixth year students did not answer better than their younger colleagues. Moreover, they completed the survey with the highest number of factual mistakes regarding the recognition of RDs from the list, and the difference between their results and those of the rest of students was considerable.

**TABLE 7:** The odds ratio of feeling prepared for caring for a patient with RD for students during the final 2 years of study.

**Odds of feeling prepared for caring for a patient with RD**			
	6th year medical students vs. 5th year medical students	5th year physiotherapy students vs. 4th year physiotherapy students	5th year nursing students vs. 4th year nursing students
OR	10.54	1.81	0.50
95%CI	2.39–46.55	0.67–4.83	0.04–5.68
*p*	**0.001**	0.12	0.29

*Statistically signifcant diferences are written in boldface.*

## Discussion

The results of this study confirm previous findings regarding the unsatisfactory level of knowledge of students of various medical faculties on RDs (Byrne, 2012; Krajnović et al., 2013; Kopeć and Podolec, 2015; Ramalle-Gómara et al., 2015; Wolyniak et al., 2015; Alam et al., 2016; Medić et al., 2016; Jonas et al., 2017; Domaradzki and Walkowiak, 2019; Walkowiak and Domaradzki, 2020). At the same time, while all the nursing, physiotherapy and medical students lacked basic information about RDs the vast majority of our respondents was aware of their knowledge deficits. Interestingly, some differences found between the groups of students were somehow surprising and difficult to explain on the basis of the individual study programs. As students from each faculty receive at least 1 year of training in genetics during the first year of their study where they study about some genetic diseases (i.e., PKU, CF, Huntington disease, sickle cell disease, Pompe disease or Niemann-Pick disease) and basic methods of genetic laboratory tests, it seems that the process of their education is rather random and knowledge about RDs is often passed casually and not in the form of a systematic academic lecture. Consequently, neither the medical nor the nursing or the physiotherapy students receive any special training in RDs.

Thus, this confirms Greb et al. (2009) observation that medical students do not retain knowledge and skills in medical genetics learned during the first years of their education. Also students who enrolled in our study received some lectures on particular types of RDs during lectures and in clinical practice, but they did not have any dedicated courses aimed particularly at RDs throughout the entire course of their studies. The only exception were 20 medical students who had chosen some elective course on metabolic diseases. Nevertheless, all the students’ knowledge about RDs was strongly dispersed and it seems that while students possibly know some individual RDs and would be able to present a simplified way of dealing with a given disease, frequently they do not identify it as a RD. Such a claim is supported by the fact that although PKU is one of the most commonly discussed RDs in medical curricula, only 42.1% of MS, 31.2% of PS and 12.4% of NS recognized it as a RD. Moreover, PKU is a model example of RD, as the one in which neonatal screening began and an effective treatment procedure was implemented.

Moreover, these results are even more intriguing as some basic information on genetics is already present in high school programs in biology, where inheritance is discussed in examples of RDs. Nevertheless, it seems that in accord with Williams’ observation (Williams, 2019), the structure and content of our current medical education is often outdated. As the current medical system is technologically orientated and market-driven, its socio-cultural aspects have become blurred (Green et al., 2002). In consequence, medical training focuses overly on scientific underpinnings, which is reinforced by the system that rewards students for recalling biomedical minutiae rather than thinking critically and holistically. This observation is exemplified by the relatively small number of medical students who believed that there should be a mandatory course on RDs in medical curricula (45.6%). Nevertheless, while such a biomedical type of training often neglects the psychosocial and humanistic aspects of patient care, it is particularly these dimensions that are required in the case of patients suffering from RDs (Williams, 2019).

Another problem is the general lack of clear guidelines and recommendations at the European Union level. Although teaching standards in medical studies are defined by the Directive 2013/55/EU of the European Parliament and of the Council amending Directive 2005/36/EC on the recognition of professional qualifications (Council of the European Union, 2013), these give only general recommendations to EU member states which shape their medical curricula and set their own examinations independently, deciding whether any mandatory or elective courses on RDs should be included in their teaching programs (McKay, 2019). Also, in Poland the Directive was implemented into the Polish legal system by the Regulation of the Minister of Science and Higher Education in 2019 and determined the standards of teaching medical professionals (Ministerstwo Nauki i Szkolnictwa Wyższego, 2019). Unfortunately, while the document is over 200 pages long, the term RD does not appear in it even once. Thus, as the expected national plan for RDs suggests the improvement of medical education on RDs, this issue needs to be re-examined. Moreover, it seems that all future healthcare professionals, including nurses, physiotherapists and physicians, should receive basic teaching about RDs, which should include information regarding the prevalence and relevance of RDs to everyday medical care, the concept of the diagnostic odyssey and possible ways of reducing it, the challenges faced by RD patients and their families and sources of information and support for RD patients. At the same time, one must acknowledge McKay’s argument that although specific RD and case studies may be used in medical curricula, teaching programs should not focus on any particular RD (McKay, 2019)—the reason being that while it is impossible to teach anyone about all 6,000–8,000 different types of RDs, we should focus on passing knowledge on the prevalence and occurrence of such diseases, patterns of dealing with patients with unusual or unknown symptoms, available sources of getting reliable information on RDs, including web pages, and raising awareness about one’s own deficits. This is of key importance because health professionals’ false beliefs in their knowledge and skills makes it difficult to change the situation of patients with rare diseases (Pisklakov et al., 2013). However, it may be worth considering Alawi’s suggestion and using RDs as teaching models, as a basis for learning fundamental principles of basic science and clinical practice (Alawi, 2019).

Yet another alarming finding was that although last year medical students differed significantly in their self-assessment of being prepared to care for RD patients, they did not answer any better than their younger colleagues did. This is of key importance, because although health professions’ self-assessment and reflecting on one’s practice can help to set appropriate learning goals and identify one’s strengths and weaknesses (Eva and Regehr, 2005), it seems that in contrast to nursing and physiotherapy students many future physicians overrated their knowledge and skills on RDs. This way of looking at one’s own competences may also be the reason for overconfidence bias, a belief universal for many professions that you know more than you really do (Croskerry, 2003). Meanwhile, such inadequate belief in one’s knowledge and skills may hinder physicians’ future professional development. Moreover, it may cause delays, misdiagnoses and lack of understanding of patients with RD, especially that research projects prove that self-evaluation and self-assessment of medical students are directly connected to the quality of provided health care (Pisklakov et al., 2013). Thus, while self-directed and continuous learning is the core concept that should be fundamental to medical education, it seems that that students should be more aware of their knowledge deficits and should recognize when to recruit additional resources: to obtain a consultation, to recruit additional support, or to refer the problem to another individual who is more competent in this domain (Eva and Regehr, 2005).

All in all, our findings confirm that just as medical curricula contain an insufficient amount of information about RDs so too nursing, physiotherapy and medical students possess inadequate knowledge and skills in this field. Moreover, as many students mistakenly believe that they are trained enough to meet the health needs of RD patients, there is an urgent need of both the improvement of medical education on RDs by including mandatory lectures and seminars into medical curricula and creating a system of postgraduate training offering specialization training sessions on RDs and raising awareness about the deficits in their training and its possible consequences for RD patients.

## Study Limitations

Simultaneously, although to our best knowledge this is one of the few studies on the knowledge on RDs among future healthcare professionals in Poland, it also has a few limitations. First, while the response rate was high, the study included students from only one medical university. Second, this study represents solely the opinions of students who agreed to participate in the study. Consequently, the results cannot be generalized for the entire population of future health professionals and more in-depth studies would be required. However, despite these limitations, some advantages of this study should also be acknowledged. Most importantly, as there is a scarcity of previous work on the topic this research helps fill the gap in the research on the knowledge of future healthcare professionals on RDs. Moreover, as it compares the knowledge of nursing, physiotherapy and medical students, it may stimulate further discussion on the need of better education not only of future physicians but also other health professionals whose role in the process of caring for RD patients is also vital.

## Conclusion

This study shows that there is a serious educational gap in future healthcare professionals about RDs. What is important is that it shows that deficits of knowledge on RDs were present not only among nursing or physiotherapy students but also future physicians. Thus, it confirms that there is an urgent need to include RDs into the medical curricula, which contain an insufficient amount of information about such diseases. Moreover, as caring for RD patients requires a special and combined approach, all healthcare specialists, including physicians, physiotherapists, nurses, midwives psychologists, dieticians or speech therapists should be enrolled in education on RDs. In order to overcome the existing knowledge deficits, it is suggested to include an RD module into the medical curricula and to implement teaching programs similar to those present in such European countries as France, Spain or the United Kingdom. Moreover, a bigger emphasis should be placed on training all medical students in basic genetics and newborn screening. Finally, while self-directed and continuous learning on RDs among healthcare professionals should be promoted, e-learning programs or courses on RDs should be also organized and Polish web pages with reliable information on RDs should be organized.

## Data Availability Statement

The raw data supporting the conclusions of this article will be made available by the authors, without undue reservation.

## Ethics Statement

The studies involving human participants were reviewed and approved by the Poznan University of Medical Sciences Bioethics Committee (1018/18). The patients/participants provided their written informed consent to participate in this study.

## Author Contributions

JD supervised conceptualization, design of the study and the collection of data. DW performed the statistical analyses. JD and DW critically revised and edited the various drafts of the manuscript and approved the final version before submission. Both authors conducted the literature search and analyses, had full access to all of the study data, discussed the results of the questionnaire, assisted in the interpretation of the data, and wrote the original draft of the manuscript.

## Conflict of Interest

The authors declare that the research was conducted in the absence of any commercial or financial relationships that could be construed as a potential conflict of interest.

## Abbreviations

RD: rare disease
EU: The European Union
PUMS: Poznan University of Medical Sciences.

## References

[B1] Alam T. Hameed A. Naveed S. Sharif N. (2016). Rare diseases: awareness amongst pharmacy students in Karachi, Pakistan. *J. Pharm. Pharm. Sci*. 4 95–101.

[B2] Alawi F. (2019). Using rare diseases as teaching models to increase awareness. *Oral Surg. Oral Med. Oral Pathol. Oral Radiol.* 128 690–691. 10.1016/j.oooo.2019.07.002 31201128

[B3] Anderson M. Elliott E. J. Zurynski Y. A. (2013). Australian families living with rare disease: experiences of diagnosis, health services use and needs for psychosocial support. *Orphanet J. Rare Dis*. 8:22. 10.1186/1750-1172-8-22 23398775 PMC3599672

[B4] Black N. Martineau F. Manacorda T. (2015). *Diagnostic Odyssey for Rare Diseases: Exploration of Potential Indicators.* London: Policy Innovation Research Unit, LSHTM.

[B5] Budych K. Helms T. M. Schultz C. (2012). How do patients with rare diseases experience the medical encounter? *Health Policy* 105 154–164. 10.1016/j.healthpol.2012.02.018 22464590

[B6] Byrne P. C. (2012). Training medical students on rare disorders. *Orphanet J. Rare Dis*. 7:A15.

[B7] Council of the European Union (2009). *Council Recommendation of 8 June 2009 on an Action in the Field of Rare Diseases (2009/C151/02).* Available online at: https://eur-lex.europa.eu/LexUriServ/LexUriServ.do?uri=OJ:C:2009:151:0007:0010:EN:PDF (accessed June 12, 2020).

[B8] Council of the European Union (2013). *Directive 2013/55/EU of the European Parliament and of the Council.* Available online at: https://eur-lex.europa.eu/legal-content/EN/TXT/HTML/?uri=CELEX:32013L0055&from=EN (accessed July 20, 2020).

[B9] Croskerry P. (2003). The importance of cognitive errors in diagnosis and strategies to minimize them. *Acad. Med*. 78 775–780. 10.1097/00001888-200308000-00003 12915363

[B10] Czech M. Baran-Kooiker A. Atikeler K. Demirtshyan M. Gaitova K. Holownia-Voloskova M. (2020). A review of rare disease policies and orphan drug reimbursement systems in 12 Eurasian countries. *Front. Public Health* 7:416. 10.3389/fpubh.2019.00416 32117845 PMC6997877

[B11] Domaradzki J. Walkowiak D. (2019). Medical students’ knowledge and opinions about rare diseases: a case study from Poland. *Intractable Rare Dis. Res*. 8 252–259. 10.5582/irdr.2019.01099 31890452 PMC6929592

[B12] Engel P. A. Bagal S. Broback M. Boice N. (2013). Physician and patient perceptions regarding physician training in rare diseases: the need for stronger educational initiatives for physicians. *J. Rare Dis*. 1 1–15.

[B13] Eurordis (2009). *The Voice of 12000 Patients. Experiences and Expectations of Rare Disease Patients on Diagnosis and Care in Europe.* Available online at: https://www.eurordis.org/IMG/pdf/voice_12000_patients/EURORDISCARE_FULLBOOKr.pdf (accessed July 3, 2020).

[B14] Eurostat (2005). The Handbook of Recommended Practices for Questionnaire Development and Testing in the European Statistical System. Available online at: https://unstats.un.org/unsd/EconStatKB/KnowledgebaseArticle10364.aspx (accessed August 12, 2020).

[B15] Eva K. W. Regehr G. (2005). Self-assessment in the health professions: a reformulation and research agenda. *Acad. Med*. 80:S46–S54. 10.1002/chp.198 16199457

[B16] Greb A. E. Brennan S. McParlane L. Page R. Bridge P. D. (2009). Retention of medical genetics knowledge and skills by medical students. *Genet. Med*. 11 365–370. 10.1097/gim.0b013e31819c6b2d 19452622

[B17] Green A. R. Carrillo J. E. Betancourt J. R. (2002). Why the disease-based model of medicine fails our patients. *West. J. Med*. 176 141–143. 11897746 PMC1071693

[B18] Jonas K. Waligóra M. Hołda M. Sulicka-Grodzicka J. Strach M. Podolec P. (2017). Knowledge *of* rare diseases among health care students – the effect of targeted education. *Przegl. Epidemiol*. 71 80–89. 28742309

[B19] Kawalec P. Sagan A. Pilc A. (2016). The correlation between HTA recommendations and reimbursement status of orphan drugs in Europe. *Orphanet J. Rare Dis.* 11:122. 10.1186/s13023-016-0501-4 27600717 PMC5012088

[B20] Khosla N. Valdez R. (2018). A compilation of national plans, policies and government actions for rare diseases in 23 countries. *Intractable Rare Dis. Res*. 7 213–222. 10.5582/irdr.2018.01085 30560012 PMC6290840

[B21] Kolasa K. Zwolinski K. M. Zah V. Zoltán K. Lewandowski T. (2018). Revealed preferences towards the appraisal of orphan drugs in Poland - multi criteria decision analysis. *Orphanet J. Rare Dis*. 13:67. 10.1186/s13023-018-0803-9 29703227 PMC5922020

[B22] Kopeć G. Podolec P. (2015). Establishing a curriculum on rare diseases for medical students. *J. Rare Cardiovasc. Dis*. 2 74–76.

[B23] Krajnović D. Arsić J. Jocić D. Milošević Georgiev A. Tasić L. Marinković V. (2013). Evaluation of pharmacists’ knowledge and attitudes regarding rare disease and orphan drugs. *Acta Med. Med*. 52 23–32. 10.5633/amm.2013.0204

[B24] Libura M. Władusiuk M. Małowicka M. Grabowska E. Gała̧zka-Sobotka M. (2016). *Gryglewicz J. Choroby Rzadkie w Polsce. Stan Obecny i Perspektywy.* Warszawa: Uczelnia Łazarskiego

[B25] McKay L. (2019). *From Amyloidosis to Zellweger Syndrome: How Can the Medical Education System Include Thousands of Rare Diseases?.* Available online at: http://www.nzmsj.com/from-amyloidosis-to-zellweger-syndrome-how-can-the-medical-education-system-include-thousands-of-rare-diseases.html. (accessed July 20, 2020).

[B26] Medić B. Divac N. Stopić B. Savić Vujović K. Glišić A. Cerovac N. (2016). The attitudes of medical students towards rare diseases: a cross-sectional study. *Vojnosanit. Pregl*. 73 703–713. 10.2298/vsp150326094m 29328568

[B27] Ministerstwo Nauki i Szkolnictwa Wyższego (2019). *Rozporza̧dzenie Ministra Nauki i Szkolnictwa Wyższego z Dnia 26 Lipca 2019 r. w Sprawie Standardów Kształcenia Przygotowuja̧cego do Wykonywania Zawodu Lekarza, Lekarza Dentysty, Farmaceuty, Pielȩgniarki, Położnej, Diagnosty Laboratoryjnego, Fizjoterapeuty i Ratownika Medycznego.* Available online at: https://prawo.sejm.gov.pl/isap.nsf/DocDetails.xsp?id=WDU20190001573 (accessed July 22, 2020).

[B28] Ministerstwo Zdrowia (2019). *Projekt Uchwały Rady Ministrów ws. Przyjȩcia Narodowego Planu dla Chorób Rzadkich.* Available online at: https://www.gov.pl/web/zdrowie/projekt-uchwaly-rady-ministrow-ws-przyjecia-narodowego-planu-dla-chorob-rzadkich (accessed July 3, 2020).

[B29] Miteva T. Jordanova R. Iskrov G. Stefanov R. (2011). General knowledge and awareness on rare diseases among general practitioners in Bulgaria. *Georgian Med. News* 193 16–19. 21617267

[B30] Moliner A. M. (2010). Creating a European Union framework for actions in the field of rare diseases. *Adv. Exp. Med. Biol*. 686 457–473. 10.1007/978-90-481-9485-8_25 20824460

[B31] Moliner A. M. Waligora J. (2017). The European Union policy in the field of rare diseases. *Adv. Exp. Med. Biol*. 1031 561–587. 10.1007/978-3-319-67144-4_30 29214592

[B32] Montserrat A. Taruscio D. (2019). Policies and actions to tackle rare diseases at European level. *Ann. Ist. Super. Sanità* 55 296–304. 31553326 10.4415/ANN_19_03_17

[B33] Pisklakov S. Rimal J. McGuirt S. (2013). Role of self-evaluation and self-assessment in medical student and resident education. *Br. J. Educ. Soc. Behav. Sci*. 4 1–9. 10.9734/bjesbs/2014/5066

[B34] Ramalle-Gómara E. Domínguez-Garrido E. Gómez-Eguílaz M. Marzo-Sola M. E. Ramón-Trapero J. L. Gil-de-Gómez J. (2020). Education and information needs for physicians about rare diseases in Spain. *Orphanet J. Rare Dis.* 15:18. 10.1186/s13023-019-1285-0 31952528 PMC6969468

[B35] Ramalle-Gómara E. Ruiz E. Quiñones C. Andrés S. Iruzubieta J. Gil-de-Gómez J. (2015). General knowledge and opinion of future health care and non-health care professionals on rare diseases. *J. Eval. Clin. Pract*. 21 198–201. 10.1111/jep.12281 25363689

[B36] Rodwell C. Aymé S. (2014). Evolution of national and European policies in the field of rare diseases and their impact over the past five years. *Orphanet J. Rare Dis*. 9:P13. 10.1186/1750-1172-9-S1-P13 PMC3942519 24580800

[B37] Rodwell C. Aymé S. (2015). Rare disease policies to improve care for patients in Europe. *Biochim. Biophys. Acta* 1852 2329–2335. 10.1016/j.bbadis.2015.02.008 25725454

[B38] Schieppati A. Henter J.-I. Daina E. Aperia A. (2008). Why rare diseases are an important medical and social issue. *Lancet* 371 2039–2041. 10.1016/s0140-6736(08)60872-7 18555915

[B39] Szegedi M. Zelei T. Arickx F. Bucsics A. Cohn-Zanchetta E. Fürst J. (2018). The European challenges of funding orphan medicinal products. *Orphanet J. Rare Dis*. 13:184. 10.1186/s13023-018-0927-y 30396361 PMC6219168

[B40] Walkowiak D. Domaradzki J. (2020). Needs assessment study of rare diseases education for nurses and nursing students in Poland. *Orphanet J. Rare Dis*. 15:167. 10.1186/s13023-020-01432-6 32600383 PMC7322909

[B41] Williams L. Z. J. (2019). Repurposing a rare opportunity: a brief insight into how implicit bias towards biomedicine impacts the care received by patients with a rare illness. *Orphanet J. Rare Dis*. 14:53. 10.1186/s13023-019-1024-6 30813964 PMC6394087

[B42] Wolyniak M. J. Bemis L. T. Prunuske A. J. (2015). Improving medical students’ knowledge of genetic disease. a review of current and emerging pedagogical practice. *Adv. Med. Educ. Pract*. 6 597–607. 10.2147/amep.s73644 26604852 PMC4629947

[B43] Zelei T. Molnárm M. J. Szegedi M. Kaló Z. (2016). Systematic review on the evaluation criteria of orphan medicines in Central and Eastern European countries. *Orphanet J. Rare Dis*. 11:72. 10.1186/s13023-016-0455-6 27259284 PMC4893267

[B44] Zurynski Y. Gonzalez A. Deverell M. Phu A. Leonard H. Christodoulou J. (2017). Rare disease: a national survey of paediatricians’ experiences and needs. *BMJ Paediatr. Open* 1:e000172. 10.1136/bmjpo-2017-000172 29637168 PMC5862166

